# The Dog in Human Society: His Great Faults and the Remedy

**Published:** 1894-01

**Authors:** Alexander Hadden

**Affiliations:** New York City


### THE DOG IN HUMAN SOCIETY: HIS GREAT FAULTS, AND THE REMEDY.

By Alexander Hadden, M.D., New York City.

The dog has been the associate and faithful servant of mankind for an indefinite period of time. His companionship can be traced quite clearly throughout the present geological period and with very conclusive evidences into the neolithic or newer sandstone. His bones have not uncommonly been found lying in company with the remains of the man of that pre-historic period.

By the people of all nations and tribes of which there are any existing history and traditions, with only a few exceptions, he has been cherished as an intimate and estimable member of their households. The followers of Zoroaster were taught to believe him to be a creation of their god Ormuzd. In their mythology he has been classed as the most faithful of creatures, and in evidence of this, the star looked upon as the sentinel and scout among the heavenly bodies, was called Sura or dog. The old Roman sportsmen regarded him as an indispensable to their circle, and as a brute companion of wonderful intelligence, activity and courage. The Greeks of immortal history cherished him, doubtless as much as we do now, as a loving pet, as a trusty guardian of person and property, and a servant of deep and never ending affections. Through Homer's Iliad they have paid a lasting tribute of their esteem to his unchanging faithfulness, in the account there given of the dog Argus and his master Ulysses.

The ancient Assyrians, too, we have reason to believe, appreciated some of his natural qualities, for they have left unmistakable evidences of it in imperishable stone. Sir H. Rawlinson says, "On the tomb of the son of Esar-Haddon, King of Assyria, is carved the form of an enormous mastiff, in the attitude of a guardian."

The ancient Egyptians, it is well authenticated, looked upon the natural instincts of the dog with superstitious reverence, did him homage, and named for him a star which they associate with the fertilizing of their fields. Their idol also, which represented sagacity and fidelity, had the head of a dog and the body of a man. Some of the Ethiopian tribes have been so marvellously impressed with his sagacity, that they have venerated him as superhuman, and others of them have thought him so superior to man in his noble qualities, that they have made him king over them. The Mohammedans, Eastern Jews and Hindoos only have ostracized him as unclean and unfit to abide in the habitations of men.

Whence this prejudice has arisen is not apparent. It cannot have been from the cry of hydrophobia, for that disease is unknown in all their countries. They tolerate him only as a public scavenger, and do not allow him to do even this humble service by day. The North American Indian regards him differently from those just mentioned. He cherishes him as a loving companion, a trusty and vigilant guard to his wigwam, and as a useful partner in his hunt for game. His attachments are so interwoven with the life of his dog that he earnestly hopes and fervently prays that the Great Spirit that rules over the living and the dead will not separate him from his boon companion when he goes to his blissful hereafter.

This sentiment was not given to these children of nature by civilization, but was found with them, and among the traditions of all their tribes when they were first discovered.

The esteem in which this creature is held by the enlightened people of our own times is best determined by what can be found in the repositories of history, legend and art, together with that which passes under our own observation. That he has been reclaimed by domestication from a wild state seems to be admitted by all writers on Natural History. This conquest is called by Baron Cuvier, the great French naturalist, the completest, most singular and most useful ever made by man. William Youatt, the celebrated writer on domestic animals, in his article on the dog, says that "he is next to the human being in the scale of intelligence, and was evidently designed to be the companion of man." The same author illustrates the inherent and singular attachments of this creature to the human family, as follows: "We exact the services of other animals; their tasks being performed, we dismiss them to their accustomed food and rest; but several varieties of the dog follow us to our homes, connect themselves with many of our pleasures and sports, and guard our sleeping hours." The same writer further remarks "that he is the only animal that is capable of disinterested affection; that he seeks to be the companion of man from spontaneous impulses alone, and that his whole aim in life seems to be to please his master, and be allowed to love and serve him."

Poets and novelists have vied with each other in making him the subject of the most soul-stirring melodies, and of stories and legends of the loftiest and tenderest sentiments. Naturalists and historians have both conclusively shown by many pages of well-authenticated facts that he has been the devoted comrade of man through all ages, periods and conditions of his existence, and is likely to be to the end of time, by reason of his varied qualities of instinct. natural endowments and incomparable fidelity. Artists of all ages, too, have joined in the plaudits of this noble and loving brute, and have reflected successfully on the canvas the sentiment that has been expressed in words, and all his peculiar attributes by which he serves and pleases mankind.

Sir Edwin Landseer has left for himself an imperishable renown in his truthful representation of this creature, by means of his brush, in his different relations to his master man. He has, by his unerring pencil, traced him in all his moral attitudes and honest longings, and has thereby preserved in form, deep and sublime emotions of joy and sorrow, on which our eyes may enjoy a never-ending feast. He has shown him to be the same faithful sentinel at the door of the peasant's cot as at that of the palace of royalty. His portrait of the noble mastiff of St. Bernard on his humane search for exhausted travellers in Alpine snowdrifts, and his masterly sketches of him as the shepherd's guard and first lieutenant, in the care and defense of flocks, and of his fatal grief on the grave of his late master, have never failed to impress the thoughtful mind that he is our Creator's special gift to man.

Sir Walter Scott, a highly distinguished student of the dog, says "he has a nature noble and incapable of deceit; that he has a share of man's intelligence, but no share of man's falsehood; and he has made historic, both in prose and verse, many of his wonderful traits and instincts." His tale in the poem Helvellyn has impressed the minds and hearts of the lovers of nature, that fidelity which follows beyond death is not exclusively human. His fondness and admiration for the dog appears very prominently in many of his writings, both prose and verse, and some of them, indeed, would be deprived of their life and spirit, were his brute companion left out. Mr. Hallam's sketch of Sir Walter at home has his dogs included, and in a short poem relating to it says:

"It was a comfort, too, to see
Those dogs that from him ne'er would rove,
And always eye him reverently
With glances of depending love."

Lord Byron's inscription on the tombstone of his Newfoundland dog gives us an insight into the profound love he bore his noble canine companion. He closes this short poetic effusion as follows:

> "Ye who perchance behold this simple urn,
> Pass on, it honors none you wish to mourn;
> To mark a friend's remains these stones arise;
> I never knew but one, and here he lies."

Cowper in his beautiful lines on the dog and the water-lily, Elizabeth Barrett Browning to Flush, my dog, Dr. Moir to his dying spaniel, J. G. Holland to his dog Blanco, and others of no less repute which I might quote, have given poetic tributes, full of admiration, tender sentiments and requited love, to their dear dumb friends and devoted servants. Both Washington Irving and Charles Dickens are said to have delighted in dogs, and to have treasured up every well-authenticated story of their wonderful instincts. Ouida, the French novelist, contributes an article to the *North American Review* on the much-despised Pomeranian or Spitz dog, and dwells especially on his peculiarities as a breed which is,—the indivisibility of his affections. It seems from her study of him that it is impossible for him to have two human beings whom he can equally love. She illustrates this by the one she owned. Others might cherish, feed—yes, even nurse, but she alone remained the whole object of his love and delight.

From the foregoing references or examples alone we might reasonably infer that the dog and human society are yet to remain inseparable, and this conclusion is based chiefly on admiration and natural attachments. But when we add to these the indispensable qualities of his instincts to many of the sports, pleasures, uses and necessities of mankind, we may positively rely upon it that he will never be eliminated from it.

One who is not an enthusiastic hunter cannot comprehend, or only in a very small way, the intrinsic value of well-trained dogs to sportsmen in their pursuit of wild fowl or animals. We can only estimate it, in part, by the fact that we cannot purchase one of them from his master for any amount of coin, unless he is a trader in these creatures. Nor can we wonder at this real attachment, when we see him quietly and fearlessly running before the gun, flushing birds, and when they are brought down, gathering them from the water and jungles without a single loss, and laying them at his master's feet in triumph. We might in some degree wonder, should we only view him connected with the more vigorous, perhaps we might say cruel sports, such as the pursuit of the fox, the stag, the wild boar or bear.

His close relations to the shepherd and general herdsman are well understood, and his indispensable services easily recognized. It is quite impossible for them to control their flocks and herds without him. Indeed these great industries both in this country and elsewhere have grown to their enormous proportions through the training into use the matchless instincts of this animal.

It has been authoritatively stated that one good, well-trained collie is worth to the shepherd a hundred men, and this, I think, will not be gainsaid, when his varied services are considered. He is the trusty sentinel who remains on duty day and night to guard the flocks against birds and beasts of prey, and becomes so well acquainted with each individual of the flock that he will gather them together when scattered, not missing one, and even separating them from the strangers, and will drive them from place to place when required with more than human care and intelligence.

Scotch history and legends are full of well-authenticated accounts of the valuable services these sagacious creatures render their master. Not only in the above mentioned instances, but also in other very perplexing and trying ordeals, such as when fogs suddenly fall upon the mountain pasture ranges, so dense that a sound misleads and the face of the sun is darkened at midday, and the shepherd is unable to discern his sheep further than a few feet distant, his dog will guard them with more than human vigilance, and keep them from scattering till the fog rises and passes away. When heavy snow storms overtake them and they fall exhausted and are banked over, he will find and save them from perishing.

These are only some of the eminent services in this domain, that this humble and loving brute renders to his master, man, and makes him indispensable to this industry and one with which he must always be identified. In the light of these services I hardly need to turn attention to anything else to establish the great importance of this animal to human society. Perhaps it may be well to add how lonely and melancholy would the poor man's home in the country be, often in a wild out of the way place, without his terrier cur which protects it by day and night, and warns off by well-expressed notices all intruders, whether they be men or animals, thus securing more safety to the life and property of the family than a paid servant. I might go on indefinitely giving instances of the pleasure and uses he affords to man, but the foregoing must suffice. Such instincts as this animal posesses, so pure and useful, have retained, and always will, the admiration and appreciation of the great part of the human family. Notwithstanding there is a mutual affection based upon natural laws existing between man and the dog, and well recognized services rendered by him to his master, there is as yet no domestic animal against which so much prejudice has been shown, or more unreasonable legislation been enacted and greater reproach been hurled. On the part of many, both in city and country, every effort has been made to ostracize him. but each attempt has signally failed. The dwelling and business places still retain their sentinels, the monks their mastiffs, the amateurs their pets, the sportsmen their companions, and the herdsmen their faithful and intelligent servants of this despised family of brutes.

The cry of hydrophobia against him, too, has frequently rent the air, and the economist has, by carefully compiled statistics, shown unwarrantable extravagance in maintaining this animal which is so very destructive to property and dangerous to life. Let us examine into the facts concerning hydrophobia and see if the causes of alarm are sufficient and worthy. This can only be properly shown by statistics, of which I think I have an abundance at hand.

In the whole United States during the year ending June 30, 1870, there were 333,169 deaths from all causes. Of these 63 only were charged to hydrophobia, and that we are to bear in mind was in a population of over 40,000,000. In the city of New York, during the year 1874, there were 28,729 deaths from all causes, 5 only from hydrophobia. During the year 1875, in New York City there were 30,709 deaths from all causes, and not any charged to hydrophobia. During the year 1876, in the same city, 29,132 deaths from all causes, and only 5 attributed to hydrophobia, and this in a population of at least 1,000,000. During the years 1872–73–74, in the city of Brooklyn, with a population of at least 500,-000, there were 13 cases of hydrophobia. Reports of the Department of Vital Statistics of New York City for the twenty-six years ending Dec. 31, 1891, give the following: 839,065 deaths from all causes; of these 53 only from hydrophobia, and of this small number all were not chargeable to the dog. During the twenty-five years ending 1872, there were in all England and Wales 1,122,072 deaths from all causes; of these, 373 were from hydrophobia. In the city of London, during the year 1874, there were only 9 deaths from hydrophobia, the population being 3,000,000.

These cases, it must be borne in mind, reported in different places and at different times, were not all chargeable to the dog, but the rats, cats and foxes played their part as well. These figures present a remarkable contrast to the degree of excitement from which whole communities suffer quite frequently from fear of dogs and hydrophobia.

I do not propose to deride the fear of this fatal poison, but I do wonder that this poison receives so much attention when far more fatal and destructive ones are even cherished in our midst. Indeed, I will say, if the proportion of deaths from hydrophobia were much greater I would wonder, for the poison that the poor sick animal implants in the human system in a moment of uncontrollable frenzy, is merciful, when compared with those which society is inflicting upon itself daily, and to which all are sensible. Where there is one victim to the poison of rabies there are thousands to many other preventable causes, such as, to overcrowded tenement houses, unsanitary school buildings, churches, street cars, and to intoxicating draughts. The poison of the former terminates with a single victim, while the latter flows into the veins of the succeeding generation.

Surely these statistical reports should convince reasonable minds that the persecution now carried on against this noble, sensitive and loving brute for this cause by local law-makers urged on by foolish fears, is almost senseless and extremely cruel

The agricultural reports are not so favorable to this animal The United States Commissioner of Agriculture gives in his report of 1865 a shocking statement of the ravages of this brute among the flocks. He gives the returns from 373 counties of 23 States of the number of sheep killed by him during the year, as follows: 77,854, and estimates the whole number killed in the United States during the same period to be about 500,000, and the loss to the raisers of sheep to be about $2,000,000.

Since 1865 no reports of these facts have been made, but there are ample reasons to believe that the ravages are no less. This offence is about the only one that can be charged against this animal. This propensity seems to be the sole remaining vice of his wild state, from which he has been no doubt to this degree, reclaimed, by domestication. He is never stimulated to this act by hunger, but does it in wild mischief.

The male dog chiefly is given to this evil, the female rarely unless in the wild state. It is also a remarkable fact that this animal never goes alone on these raids, always with a companion dog, sometimes in packs. Although one only may be in the chase, the other is near. It is also remarkable that these raids are preceded by intimate association of neighboring dogs, and it is undoubtedly out of these associations the mischief springs.

Dr. W. A. Conklin, director of the Central Park Zoological Garden, has stated to me that the raids on the sheep folds, deer enclosures, and on the swans. were always by the male dogs in packs, and in these there would often be as many as a dozen. He never knew of one dog raiding alone, and also, that in the large number that had been shot in these depredations he was quite sure that no female wȧs found. To break up the disposition of these animals to associate, and especially the preliminary association, to which referenbe has been made, should be our aim, and this is most effectually accomplished by castration, to which they should be subjected when young, about one year of age (the female or bitch should be spayed when a pup so as to lessen the danger in the operation). By this he is so changed that his attachments are for his home and for his master only, and not for those of his kind. I suggested and had this put into practice about four years ago. The animals were mongrels of good breeds, yet have made very fine and handsome watch dogs, and have no disposition to run with their kind.

One of my friends who owns a well-bred and trained shepherd dog, which he raised, when he was about a year old, had him castrated by my advice, for the purpose of breaking up his commencing tendencies to run with neighboring dogs. He reports that the operation was in every way a decided success and that his dog stays at home and is as good a herd and farm dog as one could desire. He is now four years old.

Dr. Chas. Burden, a prominent veterinary surgeon of this city, stated to me a short time ago that one of the best terriers for rats he ever knew had been castrated when quite young.

I have like testimony from others, which I might quote if necessary, but as they would illustrate the same fact, I will not proceed.

It is my implicit belief from the observation I have personally made and from evidences gathered from others on this subject, that, if no dogs were allowed to run at large except those which were emasculated, the fears and facts of hydrophobia among our people would be greatly lessened and the ravages on the flocks of sheep would cease entirely.

From the facts herein stated we may reasonably infer that the long association of the dog with human society renders the probability of his ever being cast out highly improbable.

That there is a mutual love existing between mankind and this animal is an incontrovertible fact; that he has become a necessity to many of the pleasures and vocations of the human family no one can reasonably deny. That he sometimes communicates a very fatal disease to human beings and is frequently the destroyer of valuable property are facts supported by statistics; that it is our duty to prevent this destruction of life and property, and yet retain the animal in all his usefulness should be unquestionable; that castration is a step in the right direction will be doubtless proven to be effectual by fair trial. The effect of this operation upon the bull, the horse, hog and sheep is good, and that it increases their docility, usefulness, and if intended for food, their good qualities, is indisputable, if done when of proper age, but if delayed until the animal becomes old and confirmed in his habits it certainly does not produce the desired change, but rather a tendency to become fat and lazy. It has got other advantages. He becomes, for reasons that I need not state, a less objectionable pet, far superior to the bitch, also a more reliable watch dog, as he is not likely to be decoyed away from premises he is guarding by any other dog, a stratagem that thieves and burglars often use. He will, like the castrated of other domestic animals, live to be much older, and retain his usefulness for a longer period of time. That he would be less likely to develop hydrophobia is probable, for he would be less exposed to innoculation by his association with other dogs. Castration lessens, in a marked degree, also many of his most disagreeable habits, and in no respect detracts from the useful and valuable qualities. Indeed, if any change is effected, they are essentially improved. There is a law to prevent stallions, bulls and the like from running at large. Let it be extended to the dog.

Since the reading of this paper, and the publication of the full extract in the *Forest and Stream*, June 6, 1878, many letters and notes have fallen into my hands on the subject, among them the following one, published in the *Rural New Yorkcr*, October 2, 1878, which I will here report:

"Darwin tells us in his researches into natural history that in Banda Oriental, South America, it is a common thing to see flocks of sheep, guarded by one or two dogs, at a distance of some miles from any house or man. The method of canine education thus practiced consists in separating the puppy, while very young, from its mother, and in accustoming it to its future companions. A ewe is held three or four times a day for the little thing to suck, and a nest of wool is made for it in the sheep pen. At no time is it allowed to associate with other dogs or with the children of the family. The puppy is moreover generally castrated, so that when grown up it can scarcely have any feeling in common with any of its kind. From this education it has no wish to leave the flocks, and just as another dog will defend his master, man, so will this, the sheep. On the approach of strangers the dog immediately advances barking, and the sheep all cluster in his rear as if around the oldest ram. These dogs come to the house daily for food, and when they get it skulk away as if ashamed of themselves. They can readily be taught to bring the flocks home at a certain hour in the evening. Away from the flock they seem timid with regard to other dogs, but when with their charge, neither domestic nor wild dogs dare venture to attack them."

A note from W. E. Hudson, in the *Forest and Stream*, July, 1878, calls in question my statement that bitches are not given to the vice of sheep killing. He reports that he owned two, both of which would kill sheep when the opportunity offered. Mr. J. E. writes to the *Forest and Stream*, August 1, 1878, that he owned a setter bitch that would also kill sheep These are the only instances that have come to my knowledge, and they are sufficient to shake my statement somewhat. I think, however, the vice is much less common than in the dog. His recommendation to emasculate the bitch as well as the dog I approve of, and believe the operation will greatly add to her qualities as a house pet. I have had many letters highly approving of the operation, but not one charging the castrated dog with the vice of killing sheep. I have a letter from Arthur Stevenson, of Wayne Co., Pa., who stated that he has castrated many dogs and has never known any of them to kill sheep, and thinks the animal materially improved.

